# Far-infrared suppresses skin photoaging in ultraviolet B-exposed fibroblasts and hairless mice

**DOI:** 10.1371/journal.pone.0174042

**Published:** 2017-03-16

**Authors:** Hui-Wen Chiu, Cheng-Hsien Chen, Yi-Jie Chen, Yung-Ho Hsu

**Affiliations:** 1 Division of Nephrology, Department of Internal Medicine, Shuang Ho Hospital, Taipei Medical University, New Taipei, Taiwan; 2 Graduate Institute of Clinical Medicine, College of Medicine, Taipei Medical University, Taipei, Taiwan; 3 Division of Nephrology, Department of Internal Medicine, Wan Fang Hospital, Taipei Medical University, Taipei, Taiwan; 4 Department of Internal Medicine, School of Medicine, College of Medicine, Taipei Medical University, Taipei, Taiwan; National Cheng Kung University, TAIWAN

## Abstract

Ultraviolet (UV) induces skin photoaging, which is characterized by thickening, wrinkling, pigmentation, and dryness. Collagen, which is one of the main building blocks of human skin, is regulated by collagen synthesis and collagen breakdown. Autophagy was found to block the epidermal hyperproliferative response to UVB and may play a crucial role in preventing skin photoaging. In the present study, we investigated whether far-infrared (FIR) therapy can inhibit skin photoaging via UVB irradiation in NIH 3T3 mouse embryonic fibroblasts and SKH-1 hairless mice. We found that FIR treatment significantly increased procollagen type I through the induction of the TGF-β/Smad axis. Furthermore, UVB significantly enhanced the expression of matrix metalloproteinase-1 (MMP-1) and MMP-9. FIR inhibited UVB-induced MMP-1 and MMP-9. Treatment with FIR reversed UVB-decreased type I collagen. In addition, FIR induced autophagy by inhibiting the Akt/mTOR signaling pathway. In UVB-induced skin photoaging in a hairless mouse model, FIR treatment resulted in decreased skin thickness in UVB irradiated mice and inhibited the degradation of collagen fibers. Moreover, FIR can increase procollagen type I via the inhibition of MMP-9 and induction of TGF-β in skin tissues. Therefore, our study provides evidence for the beneficial effects of FIR exposure in a model of skin photoaging.

## Introduction

Chronic exposure to ultraviolet (UV) irradiation is the major cause of skin damage that leads to premature aging of the skin, which is called photoaging. Photoaging describes the clinical signs including coarse wrinkles, roughness, laxity and pigmentation [1, 2]. Collagen, which is one of the main building blocks of human skin, is derived from fibroblasts that are regulated by both transforming growth factor-β (TGF-β), a cytokine that promotes collagen production, and activator protein-1 (AP-1), a transcription factor that promotes collagen breakdown by up regulating enzymes called matrix metalloproteinases (MMPs) [3]. The standard fibrillar collagen molecule is characterized by amino- and carboxy-terminal propeptide sequences. These form the central triple helical structure of procollagen and collagen. Three α-chains are intracellularly assembled into the triple helix following initiation of this process by the C-terminal domain. Procollagen is secreted by cells into the extracellular space and converted into collagen by the removal of the N- and C-propeptides via enzymes [4]. TGF-β induced Smad2 phosphorylation and the TGF-β/Smad axis is the main signaling pathway for collagen synthesis in dermal fibroblasts [5]. UV irradiation generates increased reactive oxygen species (ROS) levels in the skin and amplifies signals, which lead to the activation of mitogen-activated protein kinases (MAPKs) and phosphatidylinositol-3-kinase (PI3K)/Akt [6, 7]. These kinases ultimately stimulate MMPs expression and can then cause collagen degradation [8].

Autophagy is a dynamic process of degrading unnecessary or dysfunctional cell components that is activated in response to stress conditions, including starvation and misfolded protein accumulation [9–11]. Autophagy is tightly regulated by a number of pathways. The most extensively studied pathway involves Akt/mTOR, which negatively regulates autophagy [12]. mTOR plays a critical role in several signaling pathways that control cell growth, proliferation, angiogenesis, protein translation, energy homeostasis, and apoptosis [13]. UVB radiation is the major environmental risk factor for developing skin cancer, which is the most common cancer worldwide and is characterized by the aberrant activation of Akt/mTOR [14, 15]. Furthermore, the inhibition of mTOR suppresses UVB-induced keratinocyte proliferation and survival [15]. It has been reported that autophagy induction by mTOR inhibition in keratinocytes decreases proliferation. Therefore, the important roles of mTOR inhibition and autophagy provide a new target and strategy for the better prevention of UV-induced skin damage [16].

Far-infrared (FIR) radiation is an invisible electromagnetic wave with wavelengths that range from 3 to 1,000 μm according to the International Commission on Illumination. Accumulated evidence has revealed that FIR transfers energy that is perceived as heat by thermoreceptors in the surrounding skin and improves skin blood flow [17, 18]. Previous studies by us and others found that FIR has both hyperthermic effect and biological effects [18–21]. Our previous study indicated that FIR ameliorates burn-induced epidermal thickening, inflammatory cell infiltration, and the loss of distinct collagen fibers in a rat burn model. Moreover, FIR enhances autophagy and suppresses the activity of the NLRP3 inflammasome [21]. Recent evidence has shown that FIR causes collagen regeneration and the infiltration of fibroblasts that express TGF-β in wounds [19]. Of note, FIR can penetrate through skin and transfer energy into deep tissue gradually through a resonance-absorption mechanism of organic and water molecules [22]. Our recent study has demonstrated that FIR-induced promyelocytic leukemia zinc finger protein activation in vascular endothelial cells protects the vascular endothelium in diabetic mice from advanced glycation end products-induced injury [23]. Nevertheless, the biological effects of FIR on photoaging are still poorly understood. Therefore, the aim of the current study was to investigate whether FIR promotes collagen production and prevents UVB-induced collagen degradation in UVB-irradiated fibroblast cells and hairless mice. Furthermore, we analyzed whether FIR could enhance autophagy and ameliorate UVB-induced epidermal thickening.

## Materials and methods

### Cell culture

NIH 3T3 mouse embryonic fibroblasts (ATCC: CRL-1658) were obtained from the American Type Culture Collection (ATCC). The cells were cultured in Dulbecco’s modified Eagle medium (DMEM) (Gibco BRL, Grand Island, NY) supplemented with antibiotics containing 100 U/ml penicillin, 100 μg/ml streptomycin (Gibco BRL, Grand Island, NY) and 10% fetal bovine serum (HyClone, South Logan, UT, USA). The cells were incubated in a humidified atmosphere containing 5% CO_2_ at 37°C. Exponentially growing cells were detached with 0.05% trypsin-EDTA (Gibco BRL, Grand Island, NY) in DMEM.

### FIR exposure

A ceramic FIR generator, namely a WS TY301 FIR emitter (WS Far Infrared Medical Technology, Taipei, Taiwan), was used to provide the FIR exposure. This FIR emitter generates electromagnetic waves with wavelengths in the range of 3~25 μm. During the FIR exposure, an experimental group and a negative control covered with aluminum foil were set up in a culture chamber of a LiveCell^TM^ system (Pathology Devices, Westminster, MD, USA) at 37°C with a 5% CO_2_ atmosphere. The details are described in our previous study [20].

### Cell viability assay

Cellular viability was determined using the sulforhodamine B (SRB) assay. Cells were plated in 96 wells and exposed to FIR or TGF-β. After 12 or 24 h of incubation, the cells were fixed with trichloroacetic acid solution for 1 hr and SRB (Sigma Chemical Co.) was added to each well for 1 h. The plates were washed and 20 mM of Tris buffer was added. Then, the solution was read at 562 nm on an ELISA reader (Emax, Molecular Devices, Sunnyvale, CA, USA). The mean absorbance of the non-exposed cells was used as the reference value for calculating 100% cellular viability.

### Detection of collagen type I by ELISA

The culture medium of NIH 3T3 cells was collected to measure collagen type I using ELISA (MyBioSource, San Diego, CA, USA) according to the manufacturer’s instructions. The optical density of the peroxidase product was read using an ELISA reader (Emax, Molecular Devices, Sunnyvale, CA, USA) at 450 nm. Based on a standard curve, the concentration of collagen type I in each sample was determined.

### Immunofluorescence microscopy

The cells were cultured on coverslips. After FIR treatment, the cells were fixed in 4% paraformaldehyde and blocked with 1% BSA for 30 min. This was followed by incubation with a specific antibody against LC3 (MBL, Japan) for 1 h. After washing, the cells were labeled with DyLight™ 488-conjugated affinipure goat anti-rabbit IgG (Jackson ImmunoResearch Laboratories, PA, USA) for 1 h and stained with DAPI. Finally, the cells were washed in PBS, coverslipped, and examined with a confocal microscope (Leica TCS SP5).

### Western blot analysis

Total cellular protein lysates were prepared by harvesting the cells in a protein extraction buffer for 1 h at 4°C as described previously [24]. GAPDH expression was used as the protein loading control. Anti-Akt, phospho-Akt, phospho-Smad2 phospho-p70S6 K, anti-TGF-β, anti-Beclin 1 and anti-LC3 antibodies were obtained from Cell Signaling Technology (Ipswich, MA, USA). Anti-MMP-1, anti-MMP-9, anti-procollagen type 1 and anti-GAPDH antibodies were obtained from Proteintech (Rosemont, IL, USA); anti-Samd2/3 antibody was obtained from Santa Cruz (Dallas, TX, USA); and anti-p70S6 K antibody was obtained from Abcam (Cambridge, MA, USA).

### Ethics statement

All experiments on mice were performed according to the guidelines of our institute (the Guide for Care and Use of Laboratory Animals, Taipei Medical University). The animal use protocol listed below has been reviewed and approved by the Institutional Animal Care and Use Committee of Taipei Medical University, Taiwan (Approval No: LAC-2014-0283). All surgical procedure was performed under isoflurane anesthesia (Minrad Inc, PA, USA) and animals were euthanized by CO_2_ asphyxiation. All efforts were made to reduce unnecessary pain.

### UVB irradiation of mouse skin

Male hairless mice (SKH-1, 8–10 weeks old) were purchased from Charles River Laboratory (Wilmington, MA) and housed in the animal facility of Taipei Medical University. The mice were housed for at least 7 days prior to the experiments in a ventilated and temperature-controlled room and had access to water ad libitum. The mice were randomized into three treatment groups (5 mice per group): (1) normal, (2) UVB (mice were exposed to UVB and then covered with aluminum foil during the exposure to FIR for 30 min per time), and (3) UVB+FIR (mice were exposed to UVB and exposed to FIR for 30 min per time). In the UVB group, mice were exposed to 100 mJ/cm^2^ UVB radiation (one minimal erythematal dose = 100 mJ/cm^2^) five times per week for the first week and then to 200 mJ/cm^2^ three times a week for 6 weeks thereafter. In the UVB+FIR group, mice were exposed to 100 mJ/cm^2^ UVB radiation for the first week. Furthermore, the mice were exposed to 200 mJ/cm^2^ three times a week for 6 weeks UVB and exposed to FIR for 30 min five times a week. After sacrifice, some of the skin tissues were snap frozen in liquid nitrogen and stored at -80°C, and others were formalin-fixed and paraffin-embedded for immunohistochemistry.

### Histological analysis

The tissues were fixed in 10% formalin (in normal saline). After 3 days, the tissues were sectioned using a microtome and stained with hematoxylin and eosin (H&E) for histological analyses. The slides were examined microscopically and the images were recorded.

### Masson stain

The paraffin-embedded skin specimens were measured using Masson’s trichrome stain Kit (ScyTek Laboratories, Inc., UT, USA). The slides were stained with Bouin’s Fluid and Weigert’s iron hematoxylin working solution. Furthermore, the slides were differentiated in phosphomolybdic-phosphotungstic acid solution and stained with aniline blue solution. Finally, the stained skin specimens were dehydrated in series. The slides were examined microscopically and the images were recorded.

### Immunohistochemical (IHC) staining analysis

The paraffin-embedded tissue sections were dried, deparaffinized, and rehydrated. Following a microwave pretreatment in citrate buffer (pH 6.0), the slides were immersed in 3% hydrogen peroxide for 20 min to block the activity of endogenous peroxidase. After extensive washing with PBS, the slides were incubated overnight at 4°C with the anti-LC3 (MBL, Japan) or anti-MMP-9 (Proteintech, IL, USA) antibody. The sections were then incubated with the secondary antibody for 1 h at room temperature, and the slides were developed using the UltraVision Quanto HRP Detection kit (Thermo Scientific, IL, USA). Finally, the slides were counterstained using hematoxylin. Each slide was imaged.

### Statistical analysis

The data are expressed as the means ± SD. Statistical significance was determined using Student’s t-test to compare between means or one-way analysis of variance with post-hoc Dunnett’s test [25]. The differences were considered significant when p < 0.05.

## Results

### FIR increases collagen synthesis but does not cause cell proliferation in NIH 3T3 mouse embryonic fibroblasts

**Fig 1:**
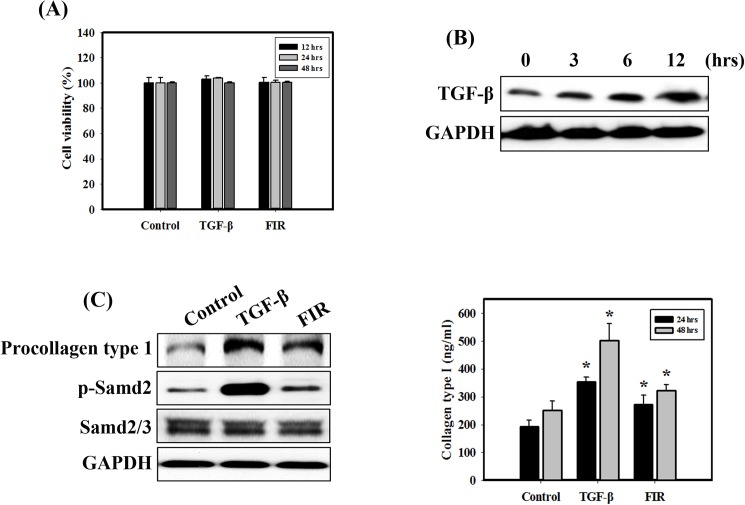
Measurement of cell viability and collagen synthesis in NIH 3T3 cells treated with FIR or TGF-β. (A) Cell viability was measured in NIH 3T3 cells treated with FIR for 30 min and cultured for 12, 24 or 48 h. Cells were treated with TGF-β (20 ng/ml) for 12, 24 or 48 h. (B) The expression levels of TGF-β protein were measured by western blot analysis following treatment with FIR. Cells were treated with FIR for 3, 6 or 12 h. (C) The expression levels of procollagen type 1, p-Smad2 and Smad2/3 proteins were measured by western blot analysis. Cells were treated with FIR for 30 min and cultured for 24 h. Cells were treated with TGF-β (20 ng/ml) for 24 h. TGF-β is a positive control for the TGF-β/Smad axis. (D) Levels of collagen type 1 in the culture medium were measured by ELISA. *p<0.05, FIR or TGF-β versus control. The data are presented as the means ± standard deviation of three independent experiments.

We investigated whether FIR affected cell viability in NIH 3T3 cells. The results showed that TGF-β and FIR treatment did not cause cell proliferation or cell death (Fig 1A). Furthermore, we determined whether FIR may increase collagen synthesis. The TGF-β/Smad axis is the main signaling pathway for collagen synthesis in fibroblasts [5]. In tendon, bone and skin, type I collagen is the major component of collagen fibrils [26]. Fig 1B shows that FIR significantly increased the expression levels of TGF-β in a time-dependent manner. Type I collagen, which is synthesized as a soluble precursor called procollagen type I, is the most abundant structural protein in the skin and connective tissue [27]. Treatment of cells with FIR and TGF-β enhanced the phosphorylation of Smad2 and procollagen type I (Fig 1C). TGF-β is a positive control for the TGF-β/Smad axis. Therefore, TGF-β treatment significantly increased the two proteins compared with FIR-exposed cells. In addition, treatment with FIR and TGF-β increased the secretion of type I collagen (Fig 1D). These results indicated that FIR treatment induced mild collagen synthesis but did not affect the viability of fibroblasts.

### FIR inhibited UVB-induced MMP-1 and MMP-9 expression and restored UVB-inhibited collagen type I

**Fig 2:**
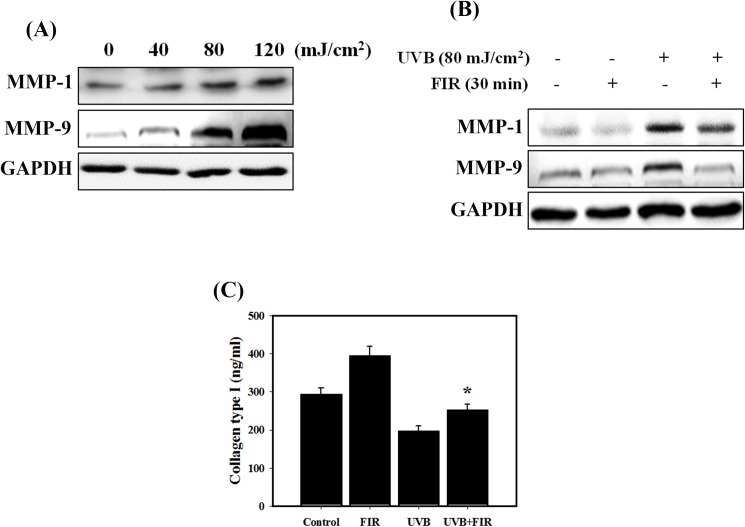
Measurement of degradation of collagen by MMPs in NIH 3T3 cells treated with UVB and/or FIR. (A) MMP-1 and MMP-9 expression in UVB-irradiated NIH 3T3 cells. Cells were irradiated with 40, 80 or 120 mJ/cm^2^ UVB for 48 h. (B) The expression levels of MMP-1 and MMP-9 proteins were measured by western blot analysis following treatment with UVB and/or FIR. Cells were irradiated with 80 mJ/cm^2^ UVB for 24 h. Then, the cells were treated with FIR for 30 min and cultured for 24 h. (C) Levels of collagen type 1 in the culture medium were measured by ELISA. Cells were irradiated with 80 mJ/cm^2^ UVB for 24 h. Then, the cells were treated with FIR for 30 min and cultured for 24 h. *p<0.05, UVB versus UVB+FIR. The data are presented as the means ± standard deviation of three independent experiments.

Skin that is damaged by UV irradiation has been shown to have elevated MMP levels, including MMP-1 (collagenase) and MMP-9 (gelatinase) [28]. In the present study, we found that UVB significantly enhanced the expression of MMP-1 and MMP-9 in NIH 3T3 cells in a dose-dependent manner (Fig 2A). Furthermore, to determine whether FIR may have a beneficial effect on UVB damaged fibroblasts, the effect of FIR on the UVB-induced MMP levels was investigated (Fig 2B). The results showed that FIR inhibited UVB-induced MMP-1 and MMP-9. In addition, FIR treatment increased secretion of type I collagen and significantly reversed UVB-decreased type I collagen (Fig 2C). These results showed that FIR treatment increased type I collagen by inhibiting MMP-1 and MMP-9.

### FIR induces autophagy by inhibiting the Akt/mTOR signaling pathway in NIH 3T3 cells

**Fig 3:**
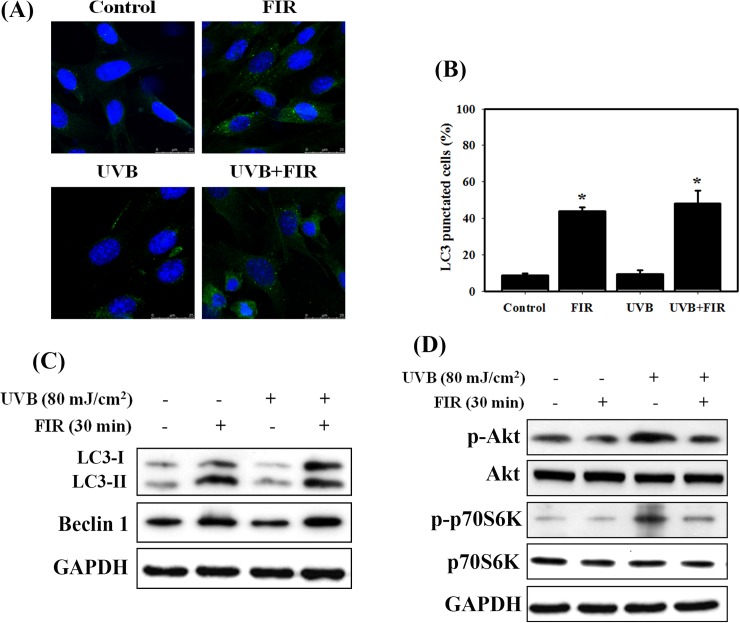
Measurement of autophagy and the Akt/mTOR signaling pathway in NIH 3T3 cells treated with UVB and/or FIR. (A) Immunofluorescence staining of LC3 protein in NIH3T3 cells treated with UVB and/or FIR. Representative cell images showing punctate LC3 distribution using a confocal microscope. Cells were irradiated with 80 mJ/cm^2^ UVB for 24 h. Then, the cells were treated with FIR for 30 min and cultured for 24 h. (B) Quantitative data calculating the percentage of LC3-positive cells. Cells were irradiated with 80 mJ/cm^2^ UVB for 24 h. Then, the cells were treated with FIR for 30 min and cultured for 24 h. *, p<0.05, versus control. The data are presented as the means ± standard deviation of three independent experiments. (C) The expression levels of autophagic-related proteins were measured by western blot analysis following treatment with UVB and FIR alone or in combination. Cells were irradiated with 80 mJ/cm^2^ UVB for 24 h. Then, the cells were treated with FIR for 30 min and cultured for 24 h. (D) The expression levels of Akt/mTOR signaling-associated proteins were measured by western blot analysis. Cells were irradiated with 80 mJ/cm^2^ UVB for 24 h. Then, the cells were treated with FIR for 30 min and cultured for 6 h.

We investigated whether FIR induced autophagy in NIH 3T3 cells (Fig 3A and 3B). Microtubule-associated protein light chain 3 (LC3) is widely used to monitor autophagy [29]. Thus, we applied confocal microscopy to determine the percentage of cells with punctate LC3 staining. The quantitative results showed a significant increase in LC3 immunopositive dots in NIH 3T3 cells that received FIR compared with control cells. We also detected the expression of autophagy-related proteins by western blotting (Fig 3C). The expression levels of LC3-II and Beclin 1 proteins increased with FIR treatment but were not affected by UVB treatment. Previous research has shown that the important pathway is Akt/mTOR, which negatively regulates autophagy [12]. We found that the phosphorylation levels of Akt and p70S6K (immediate downstream targets of mTOR) decreased in cells treated with FIR. In contrast, the levels of phosphorylated Akt and p70S6K increased in cells treated with UVB. These results indicated that FIR induced autophagy by inhibiting the Akt/mTOR signaling pathway.

### Effects of FIR on UVB-induced skin photoaging in hairless mice

**Fig 4:**
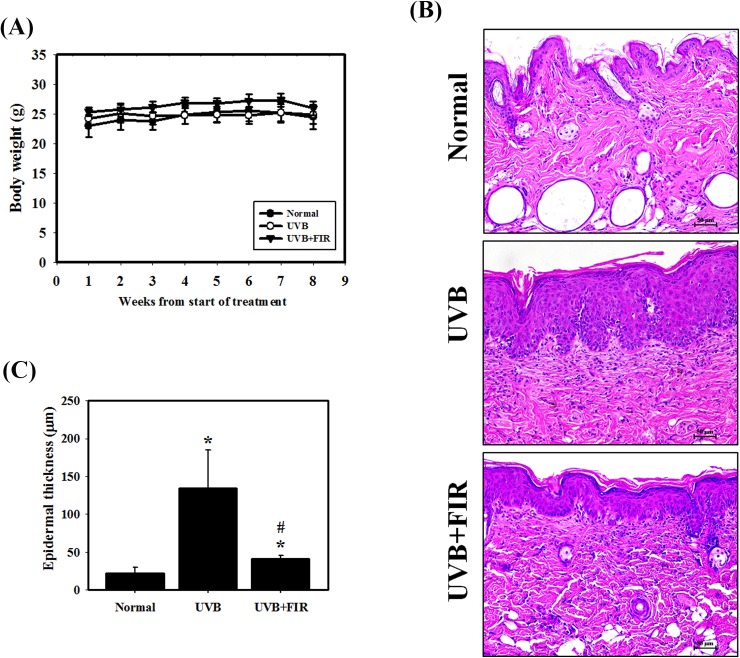
Body weight and epidermal thickness in dorsal skin of FIR-treated mice against UVB-induced skin damage. (A) Measurement of body weight in hairless mice taken once per week. (B) H&E staining and its histogram estimated for epidermal thickness. (C) Quantitative data calculating the epidermal thickness.

**Fig 5:**
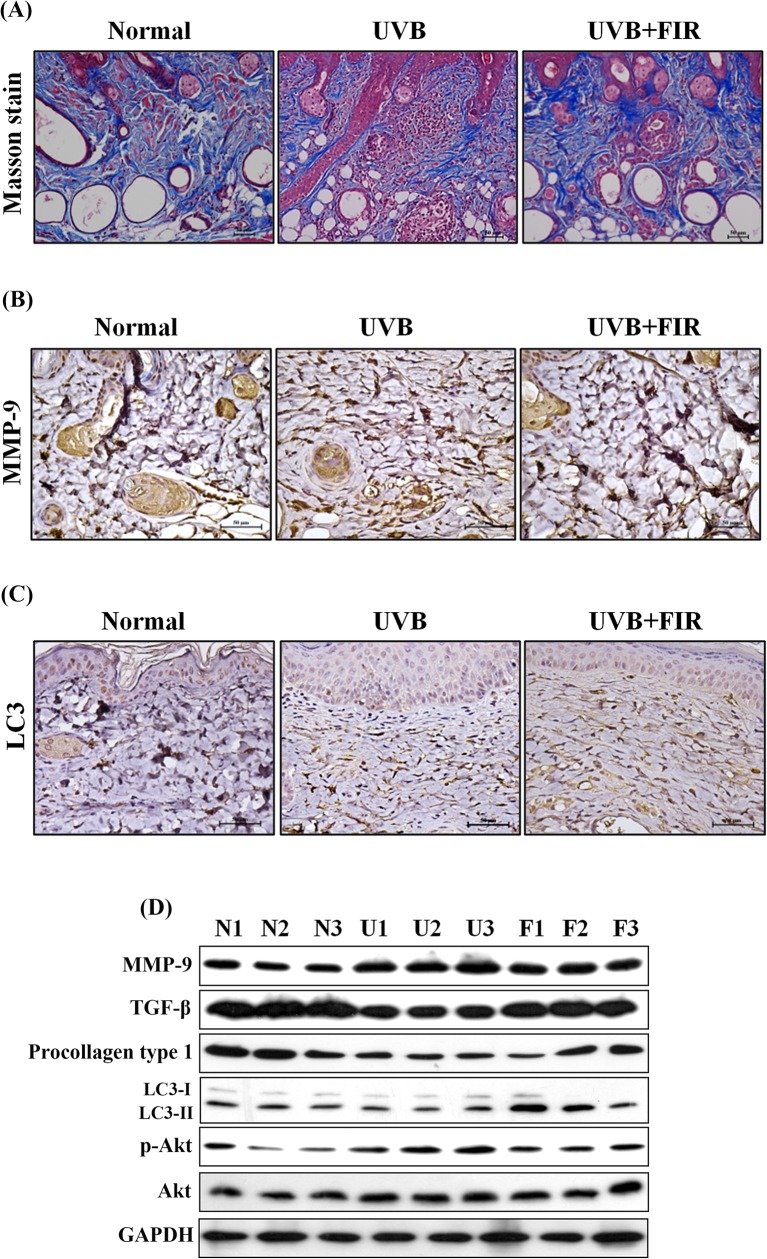
The protein expression and density of collagen fibers in a UVB-exposed hairless mouse model. (A) Masson’s trichrome staining estimated for relative collagen density. IHC staining of skin tissues was used to determine the expression levels of MMP-9 (B) and LC3 (C). (D) Western blot analysis of protein expression in skin tissues.

In UVB-induced skin photoaging in a hairless mouse model, none of the treatment regimens produced any obvious signs of toxicity in terms of the loss of body weight (Fig 4A). To investigate the effects of FIR on skin photoaging *in vivo*, hairless mice were exposed to UVB radiation. H&E staining showed the effects of FIR on histological changes of the dorsal skin (Fig 4B and 4C). As expected, UVB-irradiated mice had thicker epidermal layers than did non-irradiated mice. However, UVB-exposed and FIR-treated mice had thinner epidermal layers than mice that were exposed to UVB alone. Furthermore, Masson's trichrome stains were used to evaluate the presence and distribution of collagen. As shown in Fig 5A, the collagen fibers of UVB-irradiated mice were less dense and more erratically arranged compared to the dense, regular fibers of non-irradiated mice. We also found that the FIR group showed an increased abundance and density of collagen fibers compared with the UVB group. Next, the LC3 and MMP-9 expression levels were examined in the skin tissue using IHC staining (Fig 5B and 5C). Our results indicated that UVB irradiation induced the expression of MMP-9 but did not affect the expression of LC3 compared with the skin of non-UVB-exposed mice. A significant decrease in skin tissue that expressed MMP-9 was observed in the FIR treatment group compared with the UVB group. Additionally, FIR increased the levels of LC3 compared with UVB-exposed and normal mice. In addition to IHC staining, proteins extracted from the skin tissue were assayed by western blotting (Fig 5D). The results showed that MMP-9 and the phosphorylation of Akt were decreased and that the expression of TGF-β and procollagen type I were increased in the FIR treatment group compared with the UVB treatment group. Furthermore, the expression of LC3-II in the FIR group was higher than that in the UVB and normal groups.

## Discussion

Recently, data accumulated by us and others have revealed that FIR can be investigated as a potential therapeutic strategy in various diseases [20, 21, 30, 31]. However, the detailed mechanism is unknown. UV is the primary external stress that leads to oxidative stress, which is initiated by ROS and eventually results in premature skin aging [32]. Leung et al. found that ceramic-emitted FIR (cFIR) significantly inhibits intracellular peroxide levels and LPS-induced peroxide production by macrophages. Furthermore, cFIR blocks ROS-mediated cytotoxicity [33]. Additionally, UV is known to induce the expression of MMPs, which are the key enzymes that degrade collagen [34]. The degradation of collagen by MMPs is part of the dermal remodeling that results from skin exposure to UV. Thus, MMPs and collagen type I are attractive targets for antiphotoaging research. In the present study, FIR could suppress the UVB-induced expression of MMP-1 and MMP-9 (Fig 2). In our *in vivo* study, skin tissues from hairless mice treated with UVB showed higher MMP-9 levels than did untreated mice. Furthermore, significant decreases in the expression of MMP-9 were observed in the FIR treatment group compared with the UVB group (Fig 5B and 5D). It has been reported that the TGF-β/Smad pathway acts as a potent stimulator of the synthesis of type I collagen [5]. FIR has previously been reported to mediate therapeutic effects on skin wound healing by stimulating the secretion of TGF-β or by activating of fibroblasts [19]. Our research showed that TGF-β and phosphorylation of Smad2 protein expression increased in NIH3T3 cells following FIR treatment (Fig 1B and 1C). We also found that the expression of TGF-β and procollagen type I were increased in the FIR treatment group compared with the UVB treatment group in a model of UVB-induced skin photoaging in hairless mice (Fig 5D). Collagen fibers by Masson's trichrome stains were increased significantly in FIR-exposed mice compared with UVB-irradiated mice (Fig 5A). Previous studies have demonstrated that procollagen is translocated into the lumen of the endoplasmic reticulum (ER), in which a number of molecular chaperones and enzymes assist its folding and trimerization. Then, procollagen is secreted by cells into the extracellular space [4, 35]. In our study, we found that UVB can suppress the secretion of collagen and that FIR can significantly reverse the UVB-inhibited collagen secretion (Figs 1D and 2C). Therefore, our results showed that FIR increased collagen by inhibiting collagen breakdown and inducing collagen production. In addition, our previous study found that FIR ameliorated the burn-induced epidermal thickening [21]. Another recent study concluded that FIR pretreatment attenuates apoptosis and cell death in dehydration-stressed cultured keratinocytes through the PI3K/Akt pathway [36]. In the present study, FIR-treated mice had thinner epidermal layers than mice that were exposed to UVB alone (Fig 4B and 4C). Therefore, FIR may affect not only fibroblasts but also keratinocytes.

The PI3K/Akt/mTOR signaling pathway is essential for cell growth and proliferation. Additionally, this pathway holds in check the balance between proliferation and autophagy [37]. Previous studies have demonstrated that TGF-β promoted hepatic stellate cells activation and blocked autophagy by upregulating mTOR-p70S6K signaling [38]. However, we found that FIR can increased the expression levels of TGF-β and autophagy (Figs 1B and 3). Several studies have indicated that mTOR signaling is activated by UVB and may play an important role in skin tumorigenesis [15, 16]. Carr et al. found that rapamycin treatment or mTOR ablation inhibited UVB activation of p70S6K and blocked the epidermal hyperproliferative response to UVB [15]. Accumulated evidence has revealed that autophagy is a common consequence of mTOR inhibition. Autophagy is the major cellular pathway for the degradation of long-lived proteins and cytoplasmic organelles [16, 39]. More recently, data accumulated by us and others have revealed that FIR induces autophagy in macrophages and neuroblastoma cells [21, 31]. In the present study, FIR inhibited UVB-induced Akt/mTOR signaling both *in vitro* and *in vivo* (Figs 3D and 5D). Additionally, FIR caused statistically significant increases in autophagy in control and in UVB-treated fibroblasts (Fig 3). In the model of UVB-induced skin photoaging in hairless mice, FIR can enhance autophagy-related protein in skin tissues (Fig 5C and 5D). Previous research has shown that although a number of aging-associated pathways have been characterized, the decrease in autophagy observed in almost all aging cells and tissues is believed to be a crucial contributor to the aging phenotype and age-related diseases [40]. Zhang et al. found that miR-23a-regulated autophagy is a novel and important regulator of UV-induced premature senescence [9]. However, the role of autophagy in photoaging has not been thoroughly studied. Additionally, the underlying molecular mechanism linking autophagy to photoaging is still not known. Therefore, the molecular mechanisms that determine how autophagy affects UV-induced photoaging remain to be investigated.

**Fig 6:**
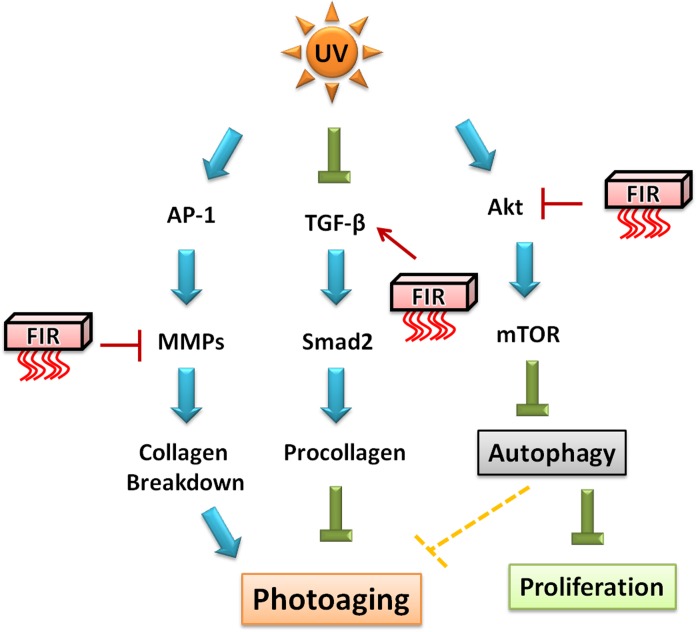
FIR pathways and effects in skin photoaging. FIR inhibits MMPs and leads to interference with collagen degradation. Furthermore, FIR increases collagen synthesis through the TGF-β/Smad pathway. In addition, FIR-induced autophagy may be mediated by inhibition of the Akt/mTOR signaling pathway. Autophagy can block the epidermal hyperproliferative response to UV and may suppress photoaging.

FIR has previously been reported to mediate therapeutic effects *in vitro* and *in vivo* on vascular endothelium and wound healing, but the potential therapeutic effects of FIR in photoaging are still unknown. To our knowledge this is the first study to report that FIR suppresses skin photoaging in UVB-exposed fibroblast and hairless mice (Fig 6). Our study provides evidence that FIR increases collagen synthesis through the TGF-β/Smad pathway. Furthermore, FIR inhibited UVB-induced MMP-1 and MMP-9 expression and restored UVB-inhibited collagen type I. In addition, FIR induces autophagy by the inhibition of Akt/mTOR signaling pathway. In UVB-induced skin photoaging in a hairless mouse model, FIR had thinner epidermal layers and an increased abundance and density of collagen fibers than mice that were exposed to UVB alone. Therefore, FIR may be a potential option in the therapeutics of skin photoaging by UVB radiation.

## References

[1] Kang S, Chung JH, Lee JH, Fisher GJ, Wan YS, Duell EA, et al Topical N-acetyl cysteine and genistein prevent ultraviolet-light-induced signaling that leads to photoaging in human skin in vivo. J Invest Dermatol. 2003;120(5):835–41. 10.1046/j.1523-1747.2003.12122.x 12713590

[2] Weiss JS, Ellis CN, Headington JT, Tincoff T, Hamilton TA, Voorhees JJ. Topical tretinoin improves photoaged skin. A double-blind vehicle-controlled study. JAMA. 1988;259(4):527–32. 3336176

[3] Mukherjee PK, Maity N, Nema NK, Sarkar BK. Bioactive compounds from natural resources against skin aging. Phytomedicine. 2011;19(1):64–73. 10.1016/j.phymed.2011.10.003 22115797

[4] Mouw JK, Ou G, Weaver VM. Extracellular matrix assembly: a multiscale deconstruction. Nat Rev Mol Cell Biol. 2014;15(12):771–85. 10.1038/nrm3902 25370693 PMC4682873

[5] Quan T, Shao Y, He T, Voorhees JJ, Fisher GJ. Reduced expression of connective tissue growth factor (CTGF/CCN2) mediates collagen loss in chronologically aged human skin. J Invest Dermatol. 2010;130(2):415–24. 10.1038/jid.2009.224 19641518 PMC2877594

[6] Kim MA, Kim HJ, Jee HJ, Kim AJ, Bae YS, Bae SS, et al Akt2, but not Akt1, is required for cell survival by inhibiting activation of JNK and p38 after UV irradiation. Oncogene. 2009;28(9):1241–7. 10.1038/onc.2008.487 19151757

[7] Scharffetter-Kochanek K, Wlaschek M, Brenneisen P, Schauen M, Blaudschun R, Wenk J. UV-induced reactive oxygen species in photocarcinogenesis and photoaging. Biol Chem. 1997;378(11):1247–57. 9426184

[8] Cimino F, Cristani M, Saija A, Bonina FP, Virgili F. Protective effects of a red orange extract on UVB-induced damage in human keratinocytes. Biofactors. 2007;30(2):129–38. 18356584 10.1002/biof.5520300206

[9] Zhang JA, Zhou BR, Xu Y, Chen X, Liu J, Gozali M, et al MiR-23a-depressed autophagy is a participant in PUVA- and UVB-induced premature senescence. Oncotarget. 2016. 10.18632/oncotarget.9357 PMC5122322 27191270

[10] Choi AM, Ryter SW, Levine B. Autophagy in human health and disease. N Engl J Med. 2013;368(19):1845–6. 10.1056/NEJMc1303158 23656658

[11] Wang M, Miller RA. Fibroblasts from long-lived mutant mice exhibit increased autophagy and lower TOR activity after nutrient deprivation or oxidative stress. Aging Cell. 2012;11(4):668–74. 10.1111/j.1474-9726.2012.00833.x 22577861 PMC3399977

[12] Yang YH, Chen K, Li B, Chen JW, Zheng XF, Wang YR, et al Estradiol inhibits osteoblast apoptosis via promotion of autophagy through the ER-ERK-mTOR pathway. Apoptosis. 2013;18(11):1363–75. 10.1007/s10495-013-0867-x 23743762

[13] Wang L, Chen L, Yu M, Xu LH, Cheng B, Lin YS, et al Discovering new mTOR inhibitors for cancer treatment through virtual screening methods and in vitro assays. Sci Rep. 2016;6:18987 10.1038/srep18987 26732172 PMC4702177

[14] Einspahr JG, Calvert V, Alberts DS, Curiel-Lewandrowski C, Warneke J, Krouse R, et al Functional protein pathway activation mapping of the progression of normal skin to squamous cell carcinoma. Cancer Prev Res (Phila). 2012;5(3):403–13. 22389437 10.1158/1940-6207.CAPR-11-0427 PMC3297971

[15] Carr TD, DiGiovanni J, Lynch CJ, Shantz LM. Inhibition of mTOR suppresses UVB-induced keratinocyte proliferation and survival. Cancer Prev Res (Phila). 2012;5(12):1394–404. 23129577 10.1158/1940-6207.CAPR-12-0272-T PMC3518591

[16] Bridgeman BB, Wang P, Ye B, Pelling JC, Volpert OV, Tong X. Inhibition of mTOR by apigenin in UVB-irradiated keratinocytes: A new implication of skin cancer prevention. Cell Signal. 2016;28(5):460–8. 10.1016/j.cellsig.2016.02.008 26876613 PMC4788564

[17] Capon A, Mordon S. Can thermal lasers promote skin wound healing? Am J Clin Dermatol. 2003;4(1):1–12. 12477368 10.2165/00128071-200304010-00001

[18] Yu SY, Chiu JH, Yang SD, Hsu YC, Lui WY, Wu CW. Biological effect of far-infrared therapy on increasing skin microcirculation in rats. Photodermatol Photoimmunol Photomed. 2006;22(2):78–86. 10.1111/j.1600-0781.2006.00208.x 16606412

[19] Toyokawa H, Matsui Y, Uhara J, Tsuchiya H, Teshima S, Nakanishi H, et al Promotive effects of far-infrared ray on full-thickness skin wound healing in rats. Exp Biol Med (Maywood). 2003;228(6):724–9. 12773705 10.1177/153537020322800612

[20] Hsu YH, Chen YC, Chen TH, Sue YM, Cheng TH, Chen JR, et al Far-infrared therapy induces the nuclear translocation of PLZF which inhibits VEGF-induced proliferation in human umbilical vein endothelial cells. PLoS One. 2012;7(1):e30674 10.1371/journal.pone.0030674 22292015 PMC3264594

[21] Chiu HW, Chen CH, Chang JN, Chen CH, Hsu YH. Far-infrared promotes burn wound healing by suppressing NLRP3 inflammasome caused by enhanced autophagy. J Mol Med (Berl). 2016;94(7):809–19. 26864306 10.1007/s00109-016-1389-0

[22] Inoue S, Kabaya M. Biological activities caused by far-infrared radiation. Int J Biometeorol. 1989;33(3):145–50. 2689357 10.1007/BF01084598

[23] Chen CH, Chen TH, Wu MY, Chou TC, Chen JR, Wei MJ, et al Far-infrared protects vascular endothelial cells from advanced glycation end products-induced injury via PLZF-mediated autophagy in diabetic mice. Sci Rep. 2017;7:40442 10.1038/srep40442 28071754 PMC5223182

[24] Chiu HW, Ho SY, Guo HR, Wang YJ. Combination treatment with arsenic trioxide and irradiation enhances autophagic effects in U118-MG cells through increased mitotic arrest and regulation of PI3K/Akt and ERK1/2 signaling pathways. Autophagy. 2009;5(4):472–83. 19242099 10.4161/auto.5.4.7759

[25] Peng PL, Kuo WH, Tseng HC, Chou FP. Synergistic tumor-killing effect of radiation and berberine combined treatment in lung cancer: the contribution of autophagic cell death. Int J Radiat Oncol Biol Phys. 2008;70(2):529–42. 10.1016/j.ijrobp.2007.08.034 18207031

[26] Fleischmajer R, Perlish JS, Timpl R, Olsen BR. Procollagen intermediates during tendon fibrillogenesis. J Histochem Cytochem. 1988;36(11):1425–32. 10.1177/36.11.3049791 3049791

[27] Makrantonaki E, Zouboulis CC. Molecular mechanisms of skin aging: state of the art. Ann N Y Acad Sci. 2007;1119:40–50. 10.1196/annals.1404.027 18056953

[28] Fisher GJ, Datta SC, Talwar HS, Wang ZQ, Varani J, Kang S, et al Molecular basis of sun-induced premature skin ageing and retinoid antagonism. Nature. 1996;379(6563):335–9. 10.1038/379335a0 8552187

[29] Mizushima N, Yoshimori T. How to interpret LC3 immunoblotting. Autophagy. 2007;3(6):542–5. 17611390 10.4161/auto.4600

[30] Wang HW, Su SH, Wang YL, Chang ST, Liao KH, Lo HH, et al MicroRNA-134 Contributes to Glucose-Induced Endothelial Cell Dysfunction and This Effect Can Be Reversed by Far-Infrared Irradiation. PLoS One. 2016;11(1):e0147067 10.1371/journal.pone.0147067 26799933 PMC4723308

[31] Chang JC, Wu SL, Hoel F, Cheng YS, Liu KH, Hsieh M, et al Far-infrared radiation protects viability in a cell model of Spinocerebellar Ataxia by preventing polyQ protein accumulation and improving mitochondrial function. Sci Rep. 2016;6:30436 10.1038/srep30436 27469193 PMC4965738

[32] Park WH. Effects of antioxidants and MAPK inhibitors on cell death and reactive oxygen species levels in H2O2-treated human pulmonary fibroblasts. Oncol Lett. 2013;5(5):1633–8. 10.3892/ol.2013.1216 23760725 PMC3678714

[33] Leung T, Lin Y, Lee C, Chen Y, Shang H, Hsiao S, et al Direct and Indirect Effects of Ceramic Far Infrared Radiation on the Hydrogen Peroxide-scavenging Capacity and on Murine Macrophages under Oxidative Stress. J Med Biol Eng. 2011;31:345–51.

[34] Vincenti MP, Brinckerhoff CE. Transcriptional regulation of collagenase (MMP-1, MMP-13) genes in arthritis: integration of complex signaling pathways for the recruitment of gene-specific transcription factors. Arthritis Res. 2002;4(3):157–64. 10.1186/ar401 12010565 PMC128926

[35] Hendershot LM, Bulleid NJ. Protein-specific chaperones: the role of hsp47 begins to gel. Curr Biol. 2000;10(24):R912–5. 11137028 10.1016/s0960-9822(00)00850-2

[36] Chen YC, Lai LC, Tu YP, Wu SD, Chen CF, Li B. Far infrared ray irradiation attenuates apoptosis and cell death of cultured keratinocytes stressed by dehydration. Journal of photochemistry and photobiology B, Biology. 2012;106:61–8. 10.1016/j.jphotobiol.2011.10.006 22062776

[37] Shimobayashi M, Hall MN. Making new contacts: the mTOR network in metabolism and signalling crosstalk. Nat Rev Mol Cell Biol. 2014;15(3):155–62. 10.1038/nrm3757 24556838

[38] Thomes PG, Brandon-Warner E, Li T, Donohue TM Jr., Schrum LW. Rev-erb agonist and TGF-beta similarly affect autophagy but differentially regulate hepatic stellate cell fibrogenic phenotype. The international journal of biochemistry & cell biology. 2016;81(Pt A):137–47. 27840152 10.1016/j.biocel.2016.11.007

[39] Levine B, Klionsky DJ. Development by self-digestion: molecular mechanisms and biological functions of autophagy. Dev Cell. 2004;6(4):463–77. 15068787 10.1016/s1534-5807(04)00099-1

[40] Rubinsztein DC, Marino G, Kroemer G. Autophagy and aging. Cell. 2011;146(5):682–95. 10.1016/j.cell.2011.07.030 21884931

